# International veterinary epilepsy task force recommendations for systematic sampling and processing of brains from epileptic dogs and cats

**DOI:** 10.1186/s12917-015-0467-9

**Published:** 2015-08-28

**Authors:** Kaspar Matiasek, Martí Pumarola i Batlle, Marco Rosati, Francisco Fernández-Flores, Andrea Fischer, Eva Wagner, Mette Berendt, Sofie F. M. Bhatti, Luisa De Risio, Robyn G. Farquhar, Sam Long, Karen Muñana, Edward E. Patterson, Akos Pakozdy, Jacques Penderis, Simon Platt, Michael Podell, Heidrun Potschka, Clare Rusbridge, Veronika M. Stein, Andrea Tipold, Holger A. Volk

**Affiliations:** Section of Clinical and Comparative Neuropathology, Centre for Clinical Veterinary Medicine, Ludwig-Maximilians-University, Veterinärstr. 13, 80539 Munich, Germany; Department of Animal Medicine and Surgery, Veterinary Faculty, Universitat Autònoma de Barcelona, Campus UAB Bellaterra, 08193 Barcelona, Spain; Department of Animal and Clinical Sciences, Faculty of Health and Medical Sciences, University of Copenhagen, Frederiksberg C, Denmark; Department of Small Animal Medicine and Clinical Biology, Faculty of Veterinary Medicine, Ghent University, Salisburylaan 133, Merelbeke, 9820 Belgium; Animal Health Trust, Lanwades Park, Kentford, Newmarket, CB8 7UU Suffolk UK; Fernside Veterinary Centre, 205 Shenley Road, Borehamwood, SG9 0TH Hertfordshire UK; University of Melbourne, 250 Princes Highway, Weibee, 3015 VIC Australia; North Carolina State University, 1052 William Moore Drive, Raleigh, NC 27607 USA; University of Minnesota College of Veterinary Medicine, D426 Veterinary Medical Center, 1352 Boyd Avenue, St. Paul, MN 55108 USA; Clinical Unit of Internal Medicine Small Animals, University of Veterinary Medicine, Veterinärplatz 1, 1210 Vienna, Austria; Vet Extra Neurology, Broadleys Veterinary Hospital, Craig Leith Road, Stirling, FK7 7LE Stirlingshire UK; College of Veterinary Medicine, University of Georgia, 501 DW Brooks Drive, Athens, GA 30602 USA; Chicago Veterinary Neurology and Neurosurgery, 3123 N. Clybourn Avenue, Chicago, IL 60618 USA; Department of Pharmacology, Toxicology and Pharmacy, Ludwig-Maximillians-University, Königinstr. 16, 80539 Munich, Germany; Fitzpatrick Referrals, Halfway Lane, Eashing, Godalming, GU7 2QQ Surrey UK; School of Veterinary Medicine, Faculty of Health and Medical Sciences, University of Surrey, Guildford, GU2 7TE Surrey UK; Department of Small Animal Medicine and Surgery, University of Veterinary Medicine Hannover, Bünteweg 9, 30559 Hannover, Germany; Department of Clinical Science and Services, Royal Veterinary College, Hatfield, AL9 7TA Hertfordshire UK

**Keywords:** Canine, Feline, Seizures, Hippocampus, Ictogenic, Epileptogenic, Processing, Neuropathology

## Abstract

Traditionally, histological investigations of the epileptic brain are required to identify epileptogenic brain lesions, to evaluate the impact of seizure activity, to search for mechanisms of drug-resistance and to look for comorbidities. For many instances, however, neuropathological studies fail to add substantial data on patients with complete clinical work-up. This may be due to sparse training in epilepsy pathology and or due to lack of neuropathological guidelines for companion animals.

The protocols introduced herein shall facilitate systematic sampling and processing of epileptic brains and therefore increase the efficacy, reliability and reproducibility of morphological studies in animals suffering from seizures.

Brain dissection protocols of two neuropathological centres with research focus in epilepsy have been optimised with regards to their diagnostic yield and accuracy, their practicability and their feasibility concerning clinical research requirements.

The recommended guidelines allow for easy, standardised and ubiquitous collection of brain regions, relevant for seizure generation. Tissues harvested the prescribed way will increase the diagnostic efficacy and provide reliable material for scientific investigations.

**Electronic supplementary material:**

The online version of this article (doi:10.1186/s12917-015-0467-9) contains supplementary material, which is available to authorized users.

## Background

Paroxysmal seizure-like events are one of the most common causes of admission to neurological services in small animal practice. With a prevalence ranging between 0.5 % and 5.0 % amongst a general non-referral population of dogs, with higher number of dogs being affected in specific breeds [1–4], epilepsy is a major health issue that severely affects the performance, cognition and behaviour of pets with recurrent seizures and thereby the quality of life of the animals and owners, the owners’ economy as well as their range of social activities [5–7].

Hence, the clinical and socioeconomical impact of epilepsy, more than its semiological and pathomechanistic resemblance to human epilepsy has been a trigger of clinical research in that field ever since. However, the most recent advances of imaging, video electroencephalography and telemetry, pharmacotherapy and neurogenetics kicked-off a new wave of enthusiasm in epileptology amongst veterinary neurologists [1, 8–13].

With some exceptions [14, 15], the pace of clinical achievements in diagnostics, classification and management of epilepsy patients in veterinary practice has not been paralleled by comparable insights into epilepsy-associated tissue changes and, in particular, those underlying drug resistance.

Brain tissue studies in clinically affected animals often are anecdotal and rarely comprise investigations for causative changes and biomarkers. If tissue studies represent the mainstay of rodent models of epilepsy, research in veterinary medicine appears to focus mainly on advancing the genetic characterisation and less so on brain pathology and anatomical changes.

One of the drawbacks that impacts negatively on the neuropathological contribution to advancing the field of canine and feline epilepsy is the lack of consensus guidelines for brain sampling, tissue processing, candidate areas, stains and algorithms. Instead, most studies employ empirical and inconsistent sampling modes and algorithms that preclude external reproducibility and therefore limit the scientific impact of the data obtained.

A standardised evaluation of brains from patients with epilepsy should provide the basis for an informed dialogue between clinicians and pathologists, and therefore requires a certain level of confidence and expertise in that specific field (Table 1).

**Table 1:** Skill level thresholds in brain pathology with special reference to epilepsy pathology

Level	Experience	Anatomical baseline skills	Semiological baseline skills & clinical neurolocalisation	Neuropathology baseline skills	Achievement
0	None:	none	none	none	n.a.
	*1* ^*st*^ *year student (veterinary & human medicine, neurobiology)*				
	*Untrained technician*				
I	Basic:	External: Recognition of cerebrum, cerebellum, brain stem and frontal/parietal/temporal/ occipital regions.	Distinction of clinical forebrain, cerebellar and brainstem signs.	Macro: Spotting malacia, gross malformations, mass lesions, haemorrhage.	Easy, Single training, Within weeks
	*2* ^*nd*^ *year student,*				
	*Trained technician*				
		Internal: Distinction of white vs grey matter.		Micro: None to basic neurohistology.	
II	Advanced:	Recognition of brain lobes, major brain regions (e.g. hippocampus thalamus, basal nuclei), tracts and of regions containing expected nuclei.	General: Specific neurolocalisation based on clinical signs.	General: Recognition of basic malformations, mass effects, haemorrhage, infiltrative lesions and basic neurodegeneration.	Demanding, Repeated training, Within months
	*Pathology & neurology residents*				
	*PhD students*				
	*General pathologist*				
			Epilepsy-specific: Distinction and localisation of seizure types.	Epilepsy-specific: Histological recognition of stereotypic seizure-associated changes.	
III	Expert:	Detailed knowledge of the species-specific topographic and functional anatomy of the brain including gyri and folia organisation, distinct nuclei, cortical areas and their patterning as well as fibre connections, neurotransmitter maps, cell markers and the vascularity.	Capable of subregional and nuclear neuro-localisation.	Recognition and classification of the above named entities, plus of microanomalies, distinct cytopathologies, brain specific disease markers and neurodegenerative disorders.	Demanding, Cont´ training, Within years
	A. broad-based				
	*Neurology-trained pathologist*				
	*Pathology-trained neurologist*				
	B. topic-based				
	*Neuroscientist*				
				Knowledge and experience in comparative neuropathology including human disorders.	

As we learned from the dichotomous evolution of epilepsy pathology in humans, the advancement of surgical therapy specifically promoted research and training in focal epilepsies and produced a diaspora of neuropathologists with exceptional skills in reading biopsies from lobectomy. Some of these diagnosticians influentially contribute to the activities of the International League Against Epilepsy (ILAE) and proved successful in implementing tissue studies to the forefront of epilepsy research [16–21].

In stark contrast, the interest in extra-focal pathologies appears generally limited and attempts to foster retrospective post-mortem analyses in human epileptics are sparse unless driven by forensic aspects [22, 23]. Naturally, in veterinary medicine pathologists most commonly face a post-mortem setting with incomplete data sets but with the fortune of the entire brain being available for examination. Due to paucity of centres with specific expertise in epilepsy pathology, however, a dedicated curriculum is difficult to acquire and experts are not easily at hand for aiding processing and evaluation of clinical cases in loco.

This limitation holds true for human autopsies as well. Most requested post-mortem examinations are conducted either by the coroner or hospital pathologists [23]. There is a general perception that neuropathologists do not necessarily have to be involved into examination of epilepsy cases until histological slides are available [23, 24]. This view bears the risk of missing essential information on the brain as prescriptions for sampling roughly propose guidance by macroscopic changes, which requires a keen eye, or from localising clinical, electrophysiological and/or imaging data, which requires special training [25].

Sending-off animal carcasses or unfixed post-mortem tissues for remote examination by specialists is impractical, expensive and, hence, not feasible. Consequently, a meaningful progress in veterinary epilepsy pathology regarding diagnosis, classification and research can be achieved only if procedures and protocols are broadly available and manageable in a para-clinical setting.

Detailed and standardised descriptions are required in particular for immediate procedures, such as harvesting of the brain, sampling from the fresh brain and fixation that can be carried out by training level 0 personnel (Table 1) but in the same vein may pose essential limits to the adjacent work-up, diagnostic yield and accuracy.

Fixed tissues do not underlie the same time pressures. Hence, investigators may acquire the neuro-anatomical knowledge necessary to sample putatively epileptogenic areas (for definition see Table 2) and those likely to carry secondary changes [23, 24] during the fixation period.

**Table 2:** Important epilepsy-related brain zones and definitions (adapted from [59])

Epileptogenic zone	Region of cortex that can generate epileptic seizures and removal or disconnection of which should lead to seizure freedom
Epileptogenic lesion	Distinct brain lesion, capable of generating and sustaining epileptic seizures
Excitable zone	Region susceptible to excitation spreading from a primary focus
Irritative zone	Region of cortex that generates interictal epileptiform discharges on EEG
Seizure/ictal onset zone	Region where a clinical seizure originates
Symptomatogenic zone	Region of cortex that generates the initial seizure presentation (signs)
Functional deficit zone	Region of cortex that in the interictal period is clinically and/or electrophysiologically abnormal
Ictal/postictal changes	Nonspecific tissue changes due to local excitotoxicity

Since “the obvious” poses the biggest obstacle to sustaining the diagnostic effort, data on the seizuring brain are poor in particular for patients with extensive structural brain lesions identified on magnetic resonance imaging (MRI), brain surgery or autopsy. It further needs to be emphasised that the trigger of epilepsy (epileptogenic lesion) and the perilesional brain tissue may not necessarily segregate or be contiguous with the perpetuating epileptogenic zone which becomes evident through incomplete seizure control after lesionectomy [1]. Restriction of the neuropathological examination to these areas, therefore, may not offer an insight into the pathobiology of an epileptic syndrome or the mechanisms of drug resistance.

Even with obvious structural lesions the diagnostician should follow the same procedures and sample the same areas as one would in cases presented with reactive epileptic seizures and idiopathic or genetic epilepsy.

Not to miss relevant information on the nature of seizures, their possible causes and consequences, and on related or unrelated comorbidities, there are three sampling schemes to consider: (1) evidence-based sampling, (2) systematic sampling and, for large brain volumes, (3) random sampling (Table 3).

**Table 3:** Neuropathological sampling schemes

Type	Determinant	Subtype	Reproducibility	Required skill levels
1	Evidence	A: structural (MRI, gross pathology)	Good	0-I
		B: functional/symptomatogenic	Good to fair	II-III
2	Systematic	A: disease-dependent, e.g. epilepsy	Good	II
		B: disease-independent	Good	I
3	Random	A: systematic (random sampling of distinct regions)	Fair to poor	I
		B: non-systematic	Poor	0

Scheme 1 comprises two different confidence levels. For obvious reasons, sampling lesions identified via MRI or on gross examination (type 1A) rarely poses a problem. In contrast, symptomatogenic approaches to brain sampling (type 1B) very much rely on both the accuracy of the neurological history (see below) and the clinical understanding of the pathologist. Inexperienced investigators go easily with scheme 1A and are inclined to sample brain regions at random (type 3A,B), regardless.

Apart from the above mentioned claim for evidence-based sampling, autopsy guidelines for epilepsy by The Royal College of Pathologists advertise simple and reproducible systematic sampling from cingulate gyrus, hippocampus, parahippocampal gyrus, middle frontal gyrus, superior and middle temporal gyri, caudate nucleus, putamen, globus pallidus, cerebellar vermis and cerebellar hemispheres [24]. This selection is based on protocols available for assessment of human neurodegenerative disorders [26] and it is expected to facilitate identification of (1) structural causes of epilepsy; (2) epilepsy-induced changes; and (3) lethal consequences of seizures, such as in Sudden Unexpected Death in Epilepsy (SUDEP) [23]. Likewise, it has been the consensus of the International Veterinary Epilepsy Task Force (IVETF) to encourage and facilitate systematic sampling of epilepsy brains in dogs and cats in order to enable standardised diagnostic approaches and to obtain tissues adequately for epilepsy research. The following protocol thus is driven by both diagnostic motives and neurobiological considerations. We hope, in particular, to facilitate studies on the involvement and role of specific brain regions for seizure propagation and semiology in dogs and cats since our current understanding derives from suspected analogies to human and rodent seizures.

Determination of a structural brain abnormality in epilepsy patients to be considered epileptogenic is based on its type, neuroanatomical localisation and seizure phenomenology. The term “epileptogenic” recently has been restricted to a set of distinctive pathologies (e.g. dysembryoplastic neuroepithelial tumours, focal cortical dysplasia, cavernoma and hippocampal sclerosis). Other pathologies more accurately are referred to as “typically epileptogenic” [2].

The fact that lesionectomy does not necessarily abolish seizures [1] should increase the awareness that the principal lesion may just elicit a process in the excitable cortex that may become an epileptogenic zone or focus itself. The area where discharges convert into clinical seizures is called seizure-onset or ictal-onset zone and may not be contiguous to the symptomatogenic zone, excitation of which determines the clinical type of seizures (Table 2).

In brain surgery of focal epilepsy, the goal is to remove the epileptogenic zone, localised by electroencephalography or functional MRI. The semiology and course, however, may be influenced by brain regions that act as seizure modifiers (e.g. claustrum) or propagators (e.g. hippocampus). Those regions should not be left unseen, even in straightforward focal structural epilepsy, to enable retrospective pathomechanistic and correlative studies. If the primary or any mirror epileptic focus cannot be excised completely, drug therapy should be continued [1].

With all understanding of required speed and efficacy of post-mortem examination as well as of ubiquitous financial constraints that affect the number of slides which can be processed, complete sampling and tissue banking constitutes the base of good research practice and of future scientific encounters that are expected to impact on the management of epileptic patients.

### Short overview of principal candidate areas

Epilepsy sampling should be guided by the acknowledgment of possible mimicry and overlap with compulsive and behavioural disorders, sleep disorders and movement disorders [3, 4]. Sampling therefore extends from the ascending reticular activating system (ARAS), via thalamocortical areas to extrapyramidal motor centres of the forebrain [5]. Little is known yet about the involvement of certain brain regions in distinct forms of canine and feline epilepsy, apart from orofacial seizures in cats [6]. Broad sampling schemes are necessary at this stage to acquire the respective data.

In most species, postictal and epileptogenic changes predominantly involve grey matter of the forebrain [5] and also Purkinje cells laden with glutamatergic synapses [7, 8]. Neurochemistry and metabolic demands determine the irritability and hence the intrinsic vulnerability to excitotoxicity. Minor local changes may translate into convulsive activity and from there spread to adjacent or remotely connected excitable areas via extra-synaptic migratory excitation or neurotransmission. Certain areas such as frontal cortex and temporal lobe are particularly susceptible to generating and perpetuating seizures and therefore should comprise the main regions of interest when sampling brain tissue [9, 10].

Amongst irritable areas, the hippocampus resembles the brain structure most commonly involved in seizures, either primarily or secondarily. Thereby, its involvement goes with essential regional, functional and interspecies differences. In kindled and pilocarpine-treated rats, for example, the ventral hippocampus presents with the earliest discharges and most extensive neuronal losses, amongst the septotemporal hippocampal axis [11, 12]. Likewise the temporoventral body (TVB), is the key area for orofacial seizures amongst temporal lobe epilepsy in cats; it is the main target of limbic encephalitis in humans and cats and it is more susceptible to hippocampal sclerosis (HS) than the dorsal parts of hippocampus [12–15].

HS is defined as pyramidal cell loss with gliosis and resembles one of the most important acquired epilepsy-promoting changes in humans [16]. It can result from necrotising and non-necrotising hippocampal lesions and thus should not be used synonymously with hippocampal necrosis. HS is subclassified according to the affected cornu ammonis segments that can be evaluated properly only in perpendicular sections of the hippocampus [17]. Currently, the high prevalence of recurrent feline epilepsy suggests a role in disease propagation in this species [13]. Its occurrence in epileptic dogs awaits further elucidation. Thus, suspected HS from hippocampal scans [18] and volumetry require to be substantiated by tissue studies [19]. Other forms of epilepsy-associated sclerosis occur in entorhinal cortex, amygdala and the subpial molecular layer [20, 21]. Their occurrence and relevance in feline and canine epilepsy remains to be clarified.

It should be noted that coexistence of HS with other epileptogenic lesions (usually outside of the hippocampus) is called “dual pathology” whereas “double pathology” refers to two epileptogenic principal lesions, other than HS [17]. If the latter occurs together with HS, this situation is referred to as “triple pathology” [22].

Depending on the cause of epilepsy and animal species, the flexure and dorsomedial tip of the hippocampal tail may contribute to the epileptic syndrome. It is important to stick to the perpendicular section throughout the longitudinal (septotemporal) axis of the hippocampus to allow for proper evaluation of the cornu ammonis (CA) segments and the dentate gyrus and for comparison in between the different hippocampal localisations. The same holds true for the subiculum and parahippocampal gyrus that may clarify whether HS is associated with reactive encephalopathy such as in hypoglycaemia [23].

Even though our insights on this topic are incomplete, temporal lobe involvement in canine epilepsy appears to differ greatly from cats [24] and predominantly affects the piriform cortex and amygdala, just rostral to the hippocampal head. Hemispheric transverse sections of the temporal lobe also allow for evaluation of entorhinal, perirhinal and postrhinal cortices, insular cortex and the claustrum, none of which has been systematically investigated in seizuring animals yet.

Being a thalamocortical syndrome, epilepsy frequently affects thalamus and lateral geniculate nucleus (own observations), which is synaptically connected to the occipital cortex. Investigation of this axis also may help to differentiate between primary versus secondary occipital lobe changes, due to forebrain enlargement and impingement by the tentorium cerebelli.

Concerning the rostral pole of the brain, the diagnostic interest in epileptic patients should carry on throughout frontal lobe rostral to lamina terminalis and include the precallosal fronto-olfactory region which is another area with low-threshold excitability.

As the frontal lobe carries the motor cortex and major extrapyramidal motor nuclei, it is the home of non-ataxic movement disorders but also resembles an important symptomatogenic zone in motor seizures with stereotypic movement pattern.

Naturally, the plethora of candidate areas for seizure development and perpetuation is intimidating. The good news is, all above mentioned areas and structures are “mutually” sampled by a rather simple trimming protocol within less than 30 min by inexperienced staff (see Additional file 1) and about 10 min by experienced investigators. Throughout all levels of expertise, regular consultation of anatomical textbooks and articles featuring topographic brain anatomy is inevitable (for useful examples see [25–28]). Thereby, the examiner needs to be aware of some terminological inconsistencies and the incompleteness of the Nomina Anatomica Veterinaria [27].

## Guidelines for brain processing

### Macro dissection and immediate post mortem procedures

**Fig. 1:**
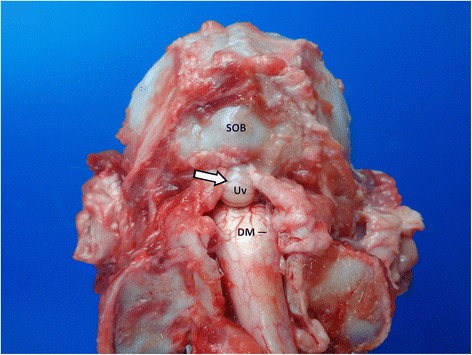
Caudodorsal view of the ventroflexed craniospinal junction in a dog after removal of paraxial muscles and laminectomie. Note the coning of the cerebellum in the foramen magnum. DM: Dura mater; SOB: supraoccipital bone; Uv: Uvula

Removal of the brain in epileptic patients employs a standard approach via removal of the skin and of the muscles of head and neck, mobilisation and dislocation of orbital contents, frontonasal osteotomy and extensive craniectomy. Before further preparation of the atlantooccipital junction, preceding decapitation or supraoccipital osteotomy, attention should be paid to possible cerebellar coning and transforaminal herniation as a consequence to intracranial pressure elevation (Fig. 1) [29].

**Fig. 2:**
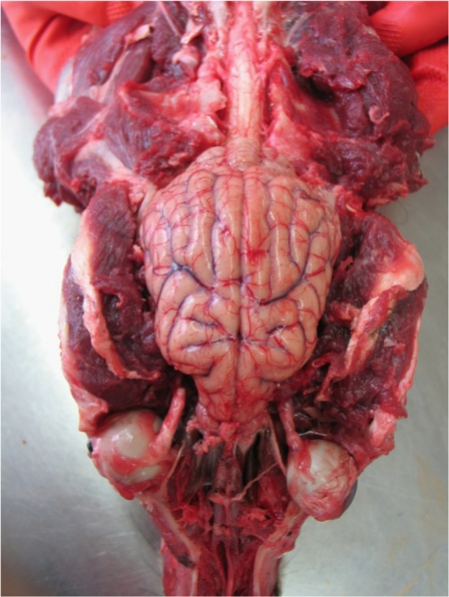
Canine brain exposed via extensive craniectomy

Upon removal of the calvaria and dorsal (mid sagittal) or ventrolateral (bilateral) durotomy, the exposed brain is inspected in situ (Fig. 2). Thereafter, the olfactory bulbs are explored and mobilised from the cribrosal lamina, the brain is lifted and cranial nerves and the pituitary stalk are transected avoiding unnecessary tearing.

The relief of having extracted the brain in one piece all too often leads to premature immersion in formalin. As a rule, a tiny piece of fresh brain tissue, deriving from a clinically or macroscopically affected target area, should be placed in RNA later® (Qiagen Inc, Hilden) or snap-frozen and stored at −80 °C for possible molecular analyses. Cerebrospinal fluid, brain swabs for culture and other case-sensitive samples for microbiological and virological testing also require to be harvested from the unfixed brain. If it comes to sampling fresh tissue for an “-omics” approach (genomic, transcriptomic, proteomic, metabolomic) to epilepsy or cryohistology, prefixation sampling protocols can be quite sophisticated and vary in accordance to the objectives of the respective study [30, 31].

If sampling is aspired from specific hippocampal regions of the autopsied brain, the dissection protocol mentioned below may apply even though the morphology is preserved better if trimmed after fixation [32]. Detection of pathological changes by less experienced staff increases significantly if gross examination is carried out on the fixed brain [32, 33]

In surgically resected epileptogenic foci, tissue is lamellated and slabs for “omics” and cryohistology are sandwiched in between slices, undergoing routine formalin-fixation and paraffin-embedding (FFPE) [32].

**Fig. 3:**
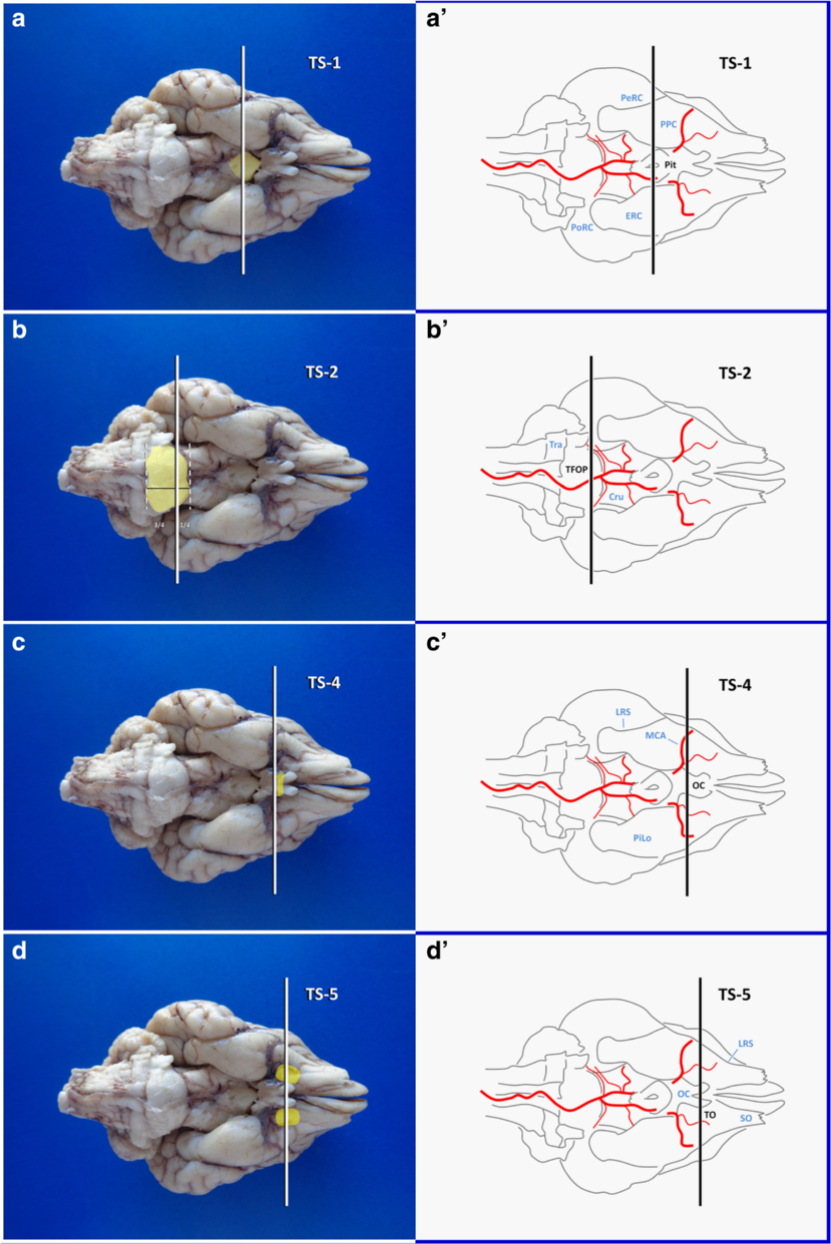
Landmarks of the ventral brain surface in a dog (Fixed brain **a**, **b**, **c**, **d**; schematic illustration **a´**, **b´**, **c´**, **d´**). Cru: crura cerebri; ERC: entorhinal cortex; LRS: lateral rhinal sulcus; MCA: middle cerebral artery; OC: optic chiasm; PeRC: perirhinal cortex; Pit: pituitary stalk; PiLo: piriform lobe; PoRC: postrhinal cortex; PPC: prepiriform cortex; SO: stria olfactoria; TFOP: transverse fibres of pons; TO: tuberculum olfactorium; Tra: trapezoid body; TS: transverse section

**Fig. 4:**
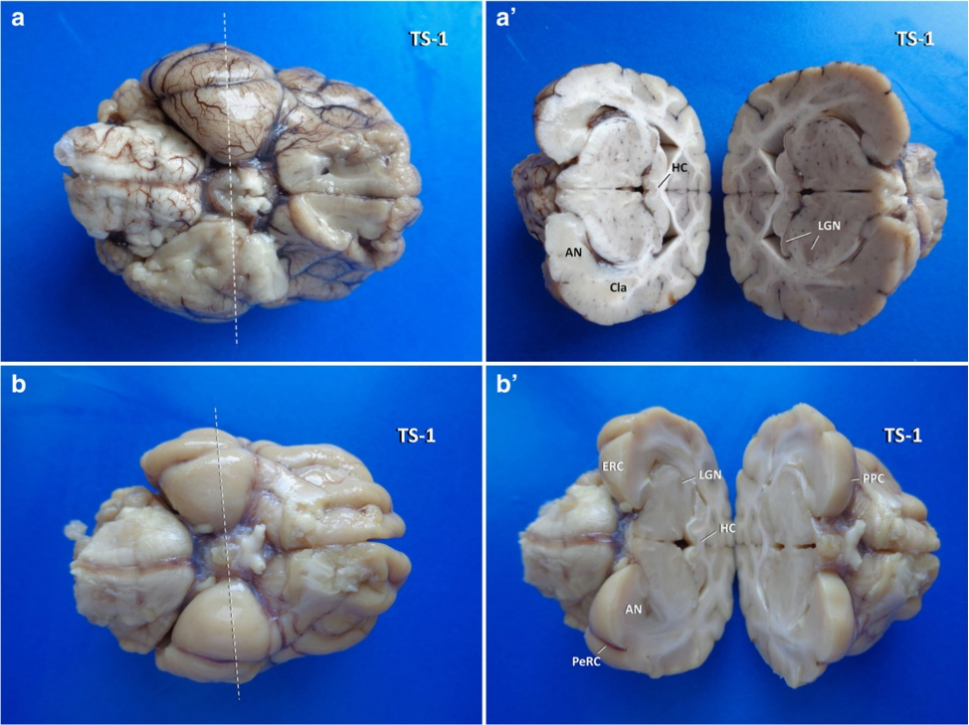
Insights into the three-dimensional orientation of the hippocampus after TS-1 (dashed line) in dog (**a**, **a´**) and cat (**b**, **b′**). AN: amygdaloid nucleus; Cla: claustrum; ERC: entorhinal cortex; HC: hippocampal commissure; LGN: lateral geniculate nucleus; PeRC: perirhinal cortex; PPC: pre-piriforme cortex

For a standard autopsy setting with an uncertain location of the epileptogenic focus, it still may be worth to snap-freeze a small section of hippocampus. Without risking the accuracy of the standard sections, mentioned below, one single transverse section at the level of infundibular recess of the third ventricle rostral to the mammillary bodies (Figs. 3 and 4) may allow for tissue-sparing identification of the dorsomedial tail of the hippocampus from which bilateral samples can easily be taken. Once, this has been achieved, the brain is immersed in a sufficient volume of 10 % neutral buffered formalin and fixed for 48 h prior to further trimming and gross examination [33].

### Post-fixation examination and trimming protocol

#### Preamble

Sampling of the different aspects of the hippocampus with sections taken perpendicularly to the longitudinal axis of the pyramidal cell band comprises the single most critical step of trimming the epileptic brain.

Nearly all other regions can be retrospectively collected and identified from fixed and trimmed pieces of brain (“bits in a bottle”). A reliable investigation of the hippocampus, however, requires both the correct angle of section and its physical connection to adjacent and connected structures such as the parahippocampal gyrus. Thus, hippocampal sampling represents the centre of efforts at this stage. However, the brain should not be cut without prior evaluation! Essential information may be missed and irreplaceably lost if macroscopic examination has been skipped.

As in a general setting, the brain should be constantly evaluated for anatomical abnormalities (Tables 4 and 5) and distinct lesions (Table 6). Concerning the untrimmed brain (Tables 4), this in particular refers to (UB-1) changes to cerebrum-cerebellum-brain stem ratio, (UB-2) abnormal brain shape and external patterning (lobes, lobules, gyri, folia), (UB-3) increased orifical width of fissures, interfolia spaces and sulci (FISS), (UB-4) leptomeningeal transparency and vascular pattern, (UB-5) changes in the rostrocerebellar space/quadrigeminal area and (UB-6) to the appearance of cranial nerve roots.

**Table 4:** Macroscopic examination of the unfixed brain

Unfixed brain—checklist		
UB-1	Changes to Cerebrum-cerebellum-brainstem size and volume ratios	
UB-2	Abnormalities of shape and patterning—tissue	
	a. lobes	
	b. lobules	
	c. gyri	
	d. folia	
UB-3	Abnormalities of shape and patterning—spaces (FISS)	
	a. fissures	
	b. sulci	
	c. interfolia spaces	
UB-4	Meningeal features	
	1. Dura mater	a. thickness/appearance
		b. venous sinuses
	2. Leptomeninx	a. transparency & thickness
		b. meningeal blood vessels
		1. filling
		2. pattern & branching
UB-5	Supracollicular features	
	1. Tentorium cerebelli	
	a. position/impingement	
	b. thickness	
	2. Perimesencephalic cisterns/rostrocerebellar space	
	3. Lamina quadrigemina	
UB-6	Cranial nerve roots	
	a. appearance	
	b. course

**Table 5:** Macroscopic examination of the trimmed brain

Trimmed brain—checklist	
TB-1	FISS base
	a. depth
	b. width
	c. course
TB-2	Cortical ribbon—subcortical white matter
	a. thickness
	b. symmetry
	c. delineation
	d. white-grey ratio
TB-3	Large white matter tracts, capsules and interposed nuclei
	a. volume & ratios
	b. symmetry
	c. delineation
	d. misplaced grey matter
TB-4	Periventricular features
	a. subependyma/glia limitans interna
	b. periventricular white matter
TB-5	Ventricular features
	a. ventricular size, symmetry, contents and communications
	b. ependymal lining and vela
	c. circumventricular organs
	d. choroid plexuses

**Table 6:** Brain lesion types

Pathological lesion category	Type & underlying pathology	
PL-1: Discolouration	Pallor	a. oedema
		b. gliosis, sclerosis, fibrosis
		c. coagulation necrosis
		d. mineralisation
		e. infiltrative disease
	Greyish	a. oedema
		b. colliquative necrosis
		c. infiltrative disease
	Yellow	a. pus
		b. caseous necrosis
		c. nuclear jaundice
	Brown	a. lipofuscinergic
		b. siderosis
	Black	a. melanin
		b. blood
	Red	blood
	Pink	carbon monoxide
PL-2: Loss & gain of tissue	Architecture sparing: disproportion & asymmetry	
	1. Gain	a. regional oedema/inflammation
		b. malformation (e.g. macrogyria)
		c. harmatoma
		d. low grade glioma
	2. Loss	a. atrophy/neurodegeneration
		b. hypoplasia
	With architectural changes	
	1. Gain	a. oedema
		b. infiltrative disease
	2. Loss	a. atrophy
		b. degeneration/necrosis
	3. Topographical	a. misplacement (e.g. heterotopia)
		b. disorganisation (dysplasia)
PL-3: Textural change	Induration	a. glial/sclerosis
		b. neoplasia
		c. fibrosis
		d. mineralisation
	Softening	a. oedema
		b. collequative necrosis
		c. inflammation
		d. neoplasia

Trimmed brain examination (Table 5), on the other hand, checklists (TB-1) course, depth and width of FISS base, (TB-2) volume, ratio, symmetry and delineation of cortical ribbon and subcortical white matter, (TB-3) visibility and symmetry of major white matter tracts and prosencephalic nuclei, (TB-4) preservation of periventricular white matter, (TB-5) appearance of the ventricular surfaces, plexuses and vela, the ventricular size, symmetry and contents.

Pathological lesions throughout the trimming process may become evident simply by (PL-1) discolouration, (PL-2) loss or gain of tissue and (PL-3) changes to the texture (Table 6).

## Specific procedures

### Trimming of the occipito-temporal region (tissue block A)

#### Orientation and planning after transverse section through the pituitary stalk or mammillary bodies

If the brain has been removed in toto, this cut (Fig. 6) should be performed with a long blade to enable fresh sampling of the dorsomedial hippocampus. It also resembles a scout section that allows for rostrocaudal localisation of the dorsomedial and ventrolateral hippocampal boundaries and of the hippocampal (syn. fornical) commissure. The insight gained from this section enables controlled sampling of the hippocampus independent of topographic variations in position and extension of the hippocampus across cats and dogs and different skull types.

In addition to providing a good overview of the middle diencephalon, this section reveals the amygdaloid nucleus that is positioned just rostral to the TVB; this should be included, as it is the second most vulnerable area for seizure-associated sclerosis, in particular in temporal lobe epilepsy identified clinically or on MRI, as well as in epilepsy patients with behavioural abnormalities and in unexplained drug resistance [34–36].

In particular in brachycephalic dogs and in cats, the ventrodorsal axis of the hippocampus is very steep and its concave plane is tilted towards the midline. Meaning that there is no way to obtain perpendicular CA sections by conventional transverse sections of the brain. The sectioning protocol should be tailored with regards to the three-dimensional placement of the hippocampus within the hemispheres (Table 7).

**Table 7:** Systematic trimming of the occipitotemporal region (Block A)

Cuts	Time	View/specimen	Landmarks and cutting levels	Orientation of sections	Aim/harvest	Difficulty
TS-1	0 min	Ventral view of whole brain	Transverse line through centre of pituitary stalk and the broadest laterolateral extension of the piriform lobe	*2D knife axis*: laterolateral.	Standard transverse section of the diencephalon.	Easy
				*Plane*: transverse.	Exposes the amygdaloid nucleus, thalamus and often lateral geniculate nucleus, piriform cortex.	
				*Blade movement*: ventrodorsal.	Allows for localisation of rostral tip of hippocampal tail and head/TVB.	
TS-2	2 min	Ventral view of brainstem	Transverse line 2 mm caudal to the rostral border of TFOP	*2D knife axis*: ventrodorsal.	Standard transverse section of the midbrain.	Easy
				*Plane*: transverse.	Prerequisite for TVB section	
				*Blade movement*: laterolateral		
TILT-1	4 min	Caudal view of occipital lobe and rostral mesencephalic stump	Horizontal line just dorsal to transverse fibres of the pons	*2D knife axis*: laterolateral.	Epilepsy-specific sections of TVB.	Easy but requires some practice
				*Plane*: oblique, transverse with rostral inclination so that the blade cuts the caudal temporal lobe flexure at right angle.	Also shows prepiriform cortex, peri/entorhinal cortex.	
				*Blade movement*: from caudoventral to rostodorsal.		
HOR-1	7 min	Same as before	Horizontal line through the upper mesencephalic aquaeduct	*2D knife axis*: laterolateral.	Epilepsy-specific section.	Easy
				*Plane*: horizontal.	Exposes CV of both hippocampi, parahippocampal gyri, postrhinal and caudal perirhinal cortex as well as lateral geniculate nucleus.	
				*Blade movement*: caudorostral.		
TILT-2L/R	9 min	Same as previous two steps	Lines perpendicularly set through the vertex of both occipitotemporal flexures.	*2D knife axis*:	Epilepsy-specific sections of both hippocampal OV and associated parahippocampal gyri, rostral colliculi, optic radiations and main visual cortices.	Easy but requires some practice
				2R: dextroventral to sinistrodorsal.		
				2L: sinistroventral to dextrodorsal		
				*Plane*: oblique, longitudinal, with lateral inclination (45° and 135°).	Also, standard procedure in transtentorial herniation.	
				*Blade movement*: caudorostral.		
TS-3	11 min	Lateral view of dorsal wedge remaining from tissue Block A	Transverse line just 1–2 mm caudal to the level of the dorsomedial tip of hippocampus.	*2D knife axis*: laterolateral.	Epilepsy-specific section of dorsomedial hippocampal tail and hippocampal commissure, corpus callosum, occipitomesial cortex including cingulate gyrus and associated subcortical white matter.	Moderately difficult as the rostrocaudal range is very small
				*Plane*: transverse.		
				*Blade movement*: dextrodorsal to sinistroventral or vice versa.		

For epilepsy-related research the following segments should be obtained bilaterally from the temporal lobe and hippocampus:

1. amygdaloid nucleus with piriform cortex;
2. temporoventral body (TVB) with entorhinal cortex;
3. caudal vertex of hippocampal flexure (CV) with post-rhinal cortex;
4. occipital vertex of hippocampal flexure (OV) with parahippocampal gyrus and visual cortex
5. dorsomedial tail at hippocampal commissure (HC) with cingulate gyrus.

Procurement of these regions is manageable for training level I personnel (Table 1) in 10 min or less if the protocol is strictly followed (Tables 7, 8, 9).

**Table 8:** Systematic trimming of the frontoparietal region (Block B)

Cuts	Time	View/specimen	Landmarks and cutting levels	Orientation of sections	Aim/harvest	Difficulty
TS-4	13 min	Ventral view of the frontal lobe	Transverse line through or just rostral to optic chiasm	*2D knife axis*: laterolateral.	Standard section of the frontal lobe.	Easy
				*Plane*: transverse.	Boundary between thalamus and basal nuclei; also shows septal nuclei, body of fornix, rostral commissure parietofrontal cortex.	
				*Blade movement*: ventrodorsal.		
TS-5	15 min	Ventral (or dorsal) view of the frontal lobe	Transverse midline section of olfactory tuberculum	*2D knife axis*: laterolateral.	Standard section of the frontal lobe providing the best view of the basal nuclei and capsules	Easy
				*Plane*: transverse.		
				*Blade movement*: ventrodorsal.		
HOR-2	17 min	Rostral view of the still connected hemispheres of olfactoryfrontal brain	2: Horizontal midline section through proreus gyrus.	*2D knife axis*: laterolateral.	Epilepsy-specific section of the susceptible olfactoryfrontal cortex.	Easy
			2′,2″:..followed by parallel sections of the ventral block with 3 mm slice thickness.	*Plane*: horizontal.	Standard section in ethmoidal pathologies.	
				*Blade movement*: rostrocaudal.		
SAG-1L/R	19 min	Rostral view of the dorsal block of the olfactoryfrontal brain	1L/R: Sagittal lines through lateral third of proreus gyrus.	*2D knife axis*: rostrocaudal.	Epilepsy-specific sections exposing motor cortex	Easy
			1 L′/R′/1 L″/R″:..followed by parallel sections with 3 mm slice thickness	*Plane*: sagittal.		
				*Blade movement*: rostrodorsal to caudoventral.

**Table 9:** Trimming and sampling of midbrain and hindbrain (Block C)

Cut	Time	View/specimen	Landmarks and cutting levels	Orientation of section	Aim/harvest	Difficulty
TS-2′	21 min	Lateral view of the midbrain	Transverse line through the intercollicular area (small brains) or caudal colliculi (large brains)	*2D knife axis*: ventrodorsal.	Standard section of the midbrain	Easy
				*Plane*: transverse.		
				*Blade movement*: laterolateral.		
TS-6	22 min	Dorsal view of the cerebellum	6: Transverse line just caudal to the primary fissure.	*2D knife axis*: laterolateral.	Standard cross section of cerebellum and medulla oblongata.	Easy
			6′:..followed by a parallel section with 3 mm slice thickness	*Plane*: transverse.	Shows central vermis, hemispheres, paraflocculus, flocculonodular lobe, cerebellar roof, caudal/middle peduncles and medulla oblongata.	
				*Blade movement*: dorsoventral along the midline axis of cerebellar hemispheres.		
TS-7	24 min	Caudodorsal view of the medullary stump	Transverse line close to the obex	*2D knife axis*: laterolateral.	Standard section of the lower brainstem.	Easy
				*Plane*: transverse.	Shows spinal tracts, vagal and associated nuclei, proprioceptive nuclei.	
				*Blade movement*: dorsoventral.		
SAG-2M	25 min	Caudal view of cerebellum and medulla	Sagittal midline section through caudal cerebellar vermis and underlying medulla.	*2D knife axis*: ventrodorsal.	Standard section of cerebellar caudal lobe and in particular useful in suspected foramen magnum herniation.	Easy
				*Plane*: sagittal, midline.		
				*Blade movement*: caudorostral.	Shows caudal vermis and midline medulla.	
SAG-2′L/R & SAG-2″L/R	27 min	Caudal view of cerebellum and medulla	2´L/R: Sagittal lines, lateral und parallel to SAG-2M on each side.	*2D knife axis*: ventrodorsal.	Standard sections for evaluation of caudal cerebellar hemispheres.	Easy
			2″L/R: ..followed by parallel sections 3 mm lateral to 2′L/R.	*Plane*: sagittal, paramedian.	Shows in particular lobules ansiformis and dorsolateral proprioceptive and vestibular areas of medulla.	
				*Blade movement*: rostrocaudal.		
SAG-3′L/R & SAG-3″L/R	29 min	Rostral view of the caudal midbrain stump and the rostral cerebellar lobe	3′L/R: Sagittal lines through the lateral boundaries of periaqueductal grey matter, about 1–2 mm lateral to the aqueduct.	*2D knife axis*: ventrodorsal	Standard sections for evaluation of rostral cerebellar lobe and in particular the effects of transtentorial herniation, as well as of pontomesencephalic transition, including caudal colliculi, leminiscus and lateral tegmental nuclei	Easy
				*Plane*: sagittal, paramedian.		
			3″L/R: ..followed by parallel sections 3 mm lateral to 3′L/R.	*Blade movement*: rostrocaudal.		

#### Procurement of the temporoventral body of the hippocampus

**Fig. 5:**
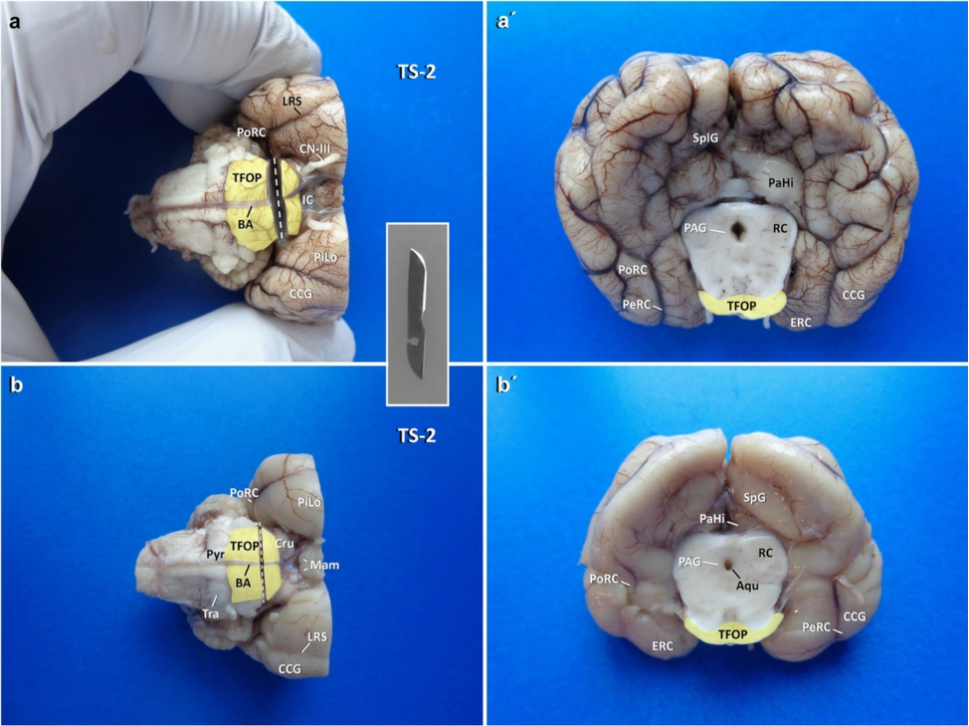
Planning of TS-2 (**a**, **b**) and inspection of the occipitotemporal brain and mesencephalon (**a′**, **b′**) in dog (**a′**, **a′**) and cat (**b**, **b′**). Transection is performed by a tipped blade (inlet). Aqu: mesencephalic aqueduct; BA: basilar artery; CCG: caudal composite gyrus; CN-III: cranial nerve III; Cru: crura cerebri; IF: intercrural cistern; LRS: lateral rhinal sulcus; Mam: mammillary bodies; PAG: periaqueductal gray matter; ParaH: parahippocampal gyrus; PeRC: perirhinal cortex; PiLo: piriform lobe; PoRC: postrhinal cortex; Pyr: pyramis. RC: rostral colliculus; SplG: splenial gyrus; TFOP: transverse fibres of pons; Tra: trapezoid body

**Fig. 6:**
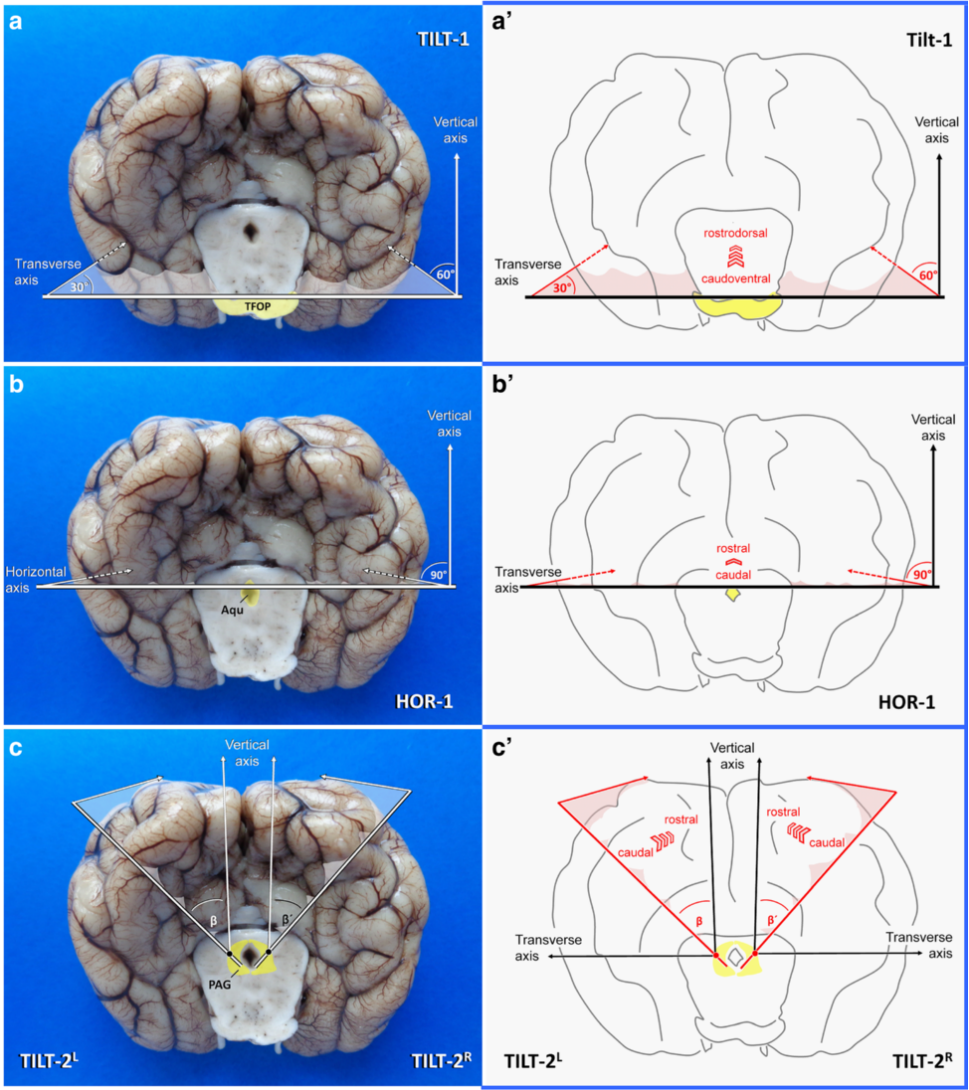
Planning of occipitotemporal brain dissection in three steps. TFOP: transverse fibres of pons; Aqu: mesencephalic aqueduct; PAG: periaqueductal gray matter. Canine brain

**Fig. 7:**
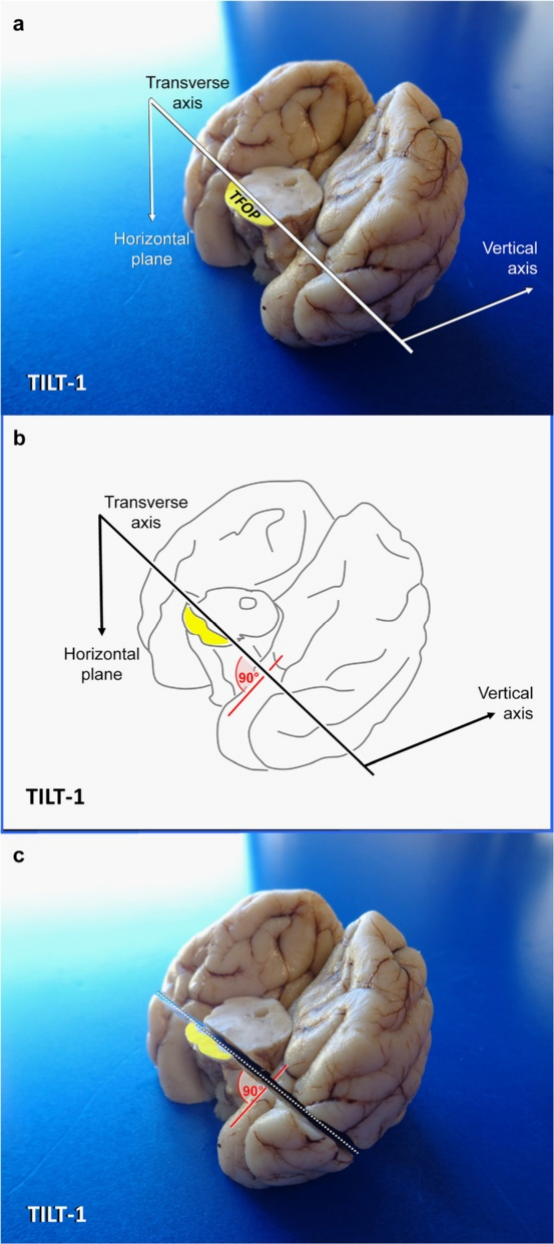
Dissection of the temporoventral body of the hippocampus via TILT-1 in a dog. MA: mesencephalic aqueduct PAG: periaqueductal grey matter; TFOP: transverse fibres of pons

For the second section (**TS-2**; Fig. 3), the caudal part of the brain is approached ventrally. The transverse fibres of the pons (TFOP) are easily recognised in between the convergence of both crura cerebri (rostral) and the origin of the pyramis (caudal). A transverse section of the brain stem is performed with a pointed blade (e.g. scalpel blades no. 11 (cats) or 22 (dogs)), pointed ventrodorsally, just separating the rostral quarter of TFOP from its caudal three quarters (Fig. 5). That way, the caudal surface of the rostral mesencephalic stump ventrally reveals the TFOP, the dorsal border of which serves as the next landmark (Figs. 6 and 7).

Insert a long blade at the horizontal laterolateral axis (0° angle), where the TFOP border the tegmentum and lower the back edge of the blade ventrally until the sharp edge points towards the caudoventral curvature of the temporal lobes (caudal composite gyrus and base of piriform lobes) at a right angle (Fig. 7).

If you perform the section in this tilted caudoventral to rostrodorsal fashion (**TILT-1**), you will create a perpendicular section of the entorhinal cortex and TVB; differential evaluation of individual CA segments (e.g. for HS) or evaluation of the dentate gyrus and subiculum pathology will be easy and reliable.

Adequate slices will be ready to be put in standard cassettes after another section is made parallel to the surface of the wedge (**TILT-1′**) and a longitudinal cut is made through the attached brain stem (see Additional file 1).

#### Obtainment of the caudal vertex of hippocampal flexure

At the level of lateral geniculate nuclei (LGN), MR investigation of the hippocampus in angled horizontal plane (or coronal in humans) may allow for assessment of hippocampal atrophy and HS [37]. Even though histopathological changes usually are more advanced in TVB, this adjacent region should be sampled for correlative investigations and for changes to the postrhinal and perirhinal cortices [38–40].

**Fig. 8:**
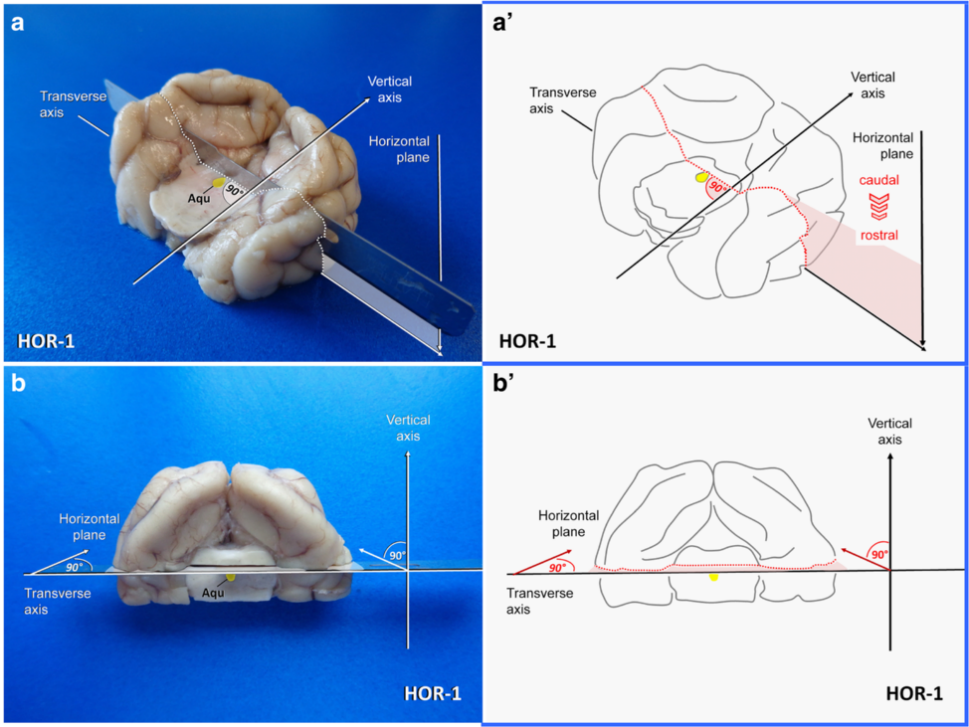
Dissection of the caudal vertex of hippocampus via HOR-1 in a dog illustrated before (**a**, **a´**) and after (**b**, **b´**) procurement of the temporoventral body. Aqu: mesencephalic aqueduct

It can be easily approached from caudal aspect again (Fig. 6). A long blade is positioned horizontally at the dorsal border of the mesencephalic aqueduct (Fig. 8). This section (**HOR-1**) simply is conducted perpendicular to the transectional surface of the mesencephalic stump in a caudorostral fashion (horizontal plane). If the level has been correctly chosen, the LGN are seen just opposite to the hippocampi at the other side of the choroidal fissure (Fig. 10).

#### Procurement of the occipital vertex of hippocampal flexure

Additional sections of brain block A allow for a contextual evaluation of the hippocampal OV, the parahippocampal and splenial gyri, both directly exposed to the tentorium and, hence, prone to impingement during herniation [29].

**Fig. 9:**
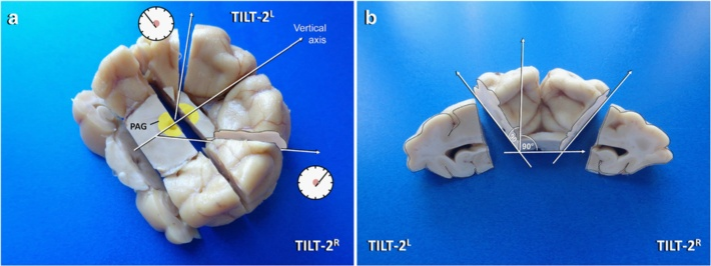
Dissection (**a**) and inspection (**b**, dashed line) of the occipital vertex of the hippocampus in a dog. PAG: periaqueductal gray matter

On caudal view of the left occipital lobe, the blade is directed rostrally while the knife points clockwise to 10.30 and the pivot is set slightly left to mesencephalic aequeduct, where the periaquaeductal grey matter dorsolaterally is expected to border the tegmentum (**TILT-2**^L^ Fig. 6; Fig. 9).

That way, the blade is supposed to cut the parahippocampal gyrus and hippocampus perpendicularly. For the right hemisphere the procedure is repeated just mirror inverted (**TILT-2R**; Figs. 6 and 9).

#### Procurement of the dorsomedial hippocampal tail and hippocampal commissure

Longitudinal variations of pathological lesions along the septotemporal axis are frequently seen but have been rarely associated to distinct aetiologies. Exceptions are toxicopathological studies and rodent models of epilepsy [11]. Respecting the varying connectivities, functions and metabolism, and in particular our lack of knowledge regarding selective vulnerabilities and involvement, the dorsomedial hippocampus should not be omitted.

**Fig. 10:**
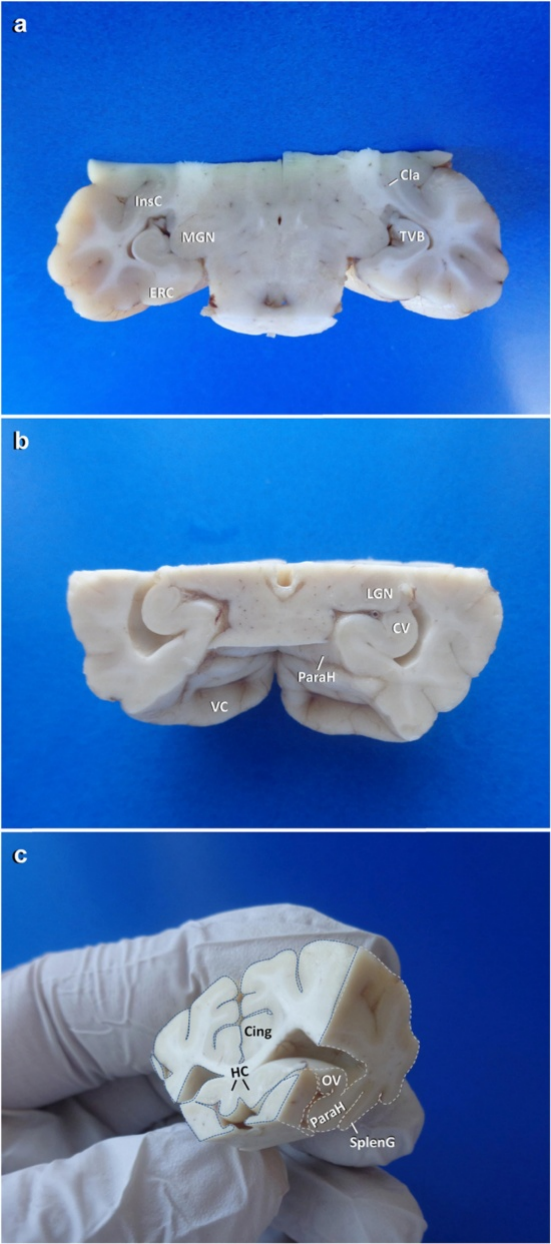
Overview of dissected temporoventral body (**a**: TVB), caudal vertex (**b**: CV), occipital vertex (**c**: OV) and commissure of hippocampus (**c**: HC). Cing: cingulate gyrus; Cla: claustrum; ERC: entorhinal cortex; InsC: insular cortex; LGN: lateral geniculate nucleus; MGN: medial geniculate nucleus; ParaH: parahippocampal gyrus; SplG: splenial gyrus; VC: visual cortex

After obtainment of the occipital vertices, a wedge shaped piece of block A remains containing the occipitomesial cortex, marginal and ectomarginal gyri bilaterally. Rostral inspection of this wedge allows for judgement of the rostral tip of the hippocampal tail in the midline, ventrally attached to the fornix. A transverse section (**TS-3**) should be performed just about 1 mm caudal to this point. This level usually provides a perpendicular view of the dorsal CA segments and DG and of the hippocampal commissure (Figs. 4 and 10) that may be one of the pathways wiring excitations to the contralateral side of the brain.

**Fig. 11:**
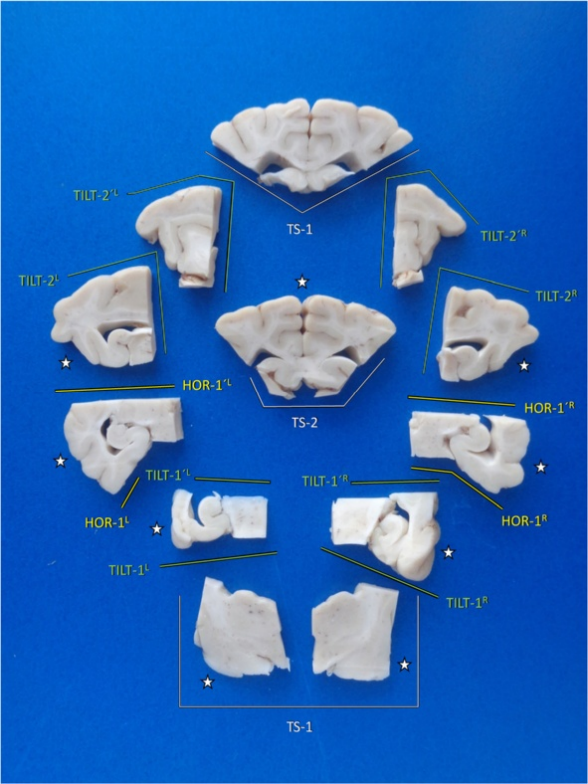
Overview of main brain slabs of Block A in correct angle of section. A selection of these may be further processed for histology. Asterisks mark our recommendation for systematic epilepsy pathology studies

A survey on the brain slides possibly sampled by trimming of tissue block A is provided in Fig. 11.

### Trimming of the parieto-frontal region (tissue block B)

**Fig. 12:**
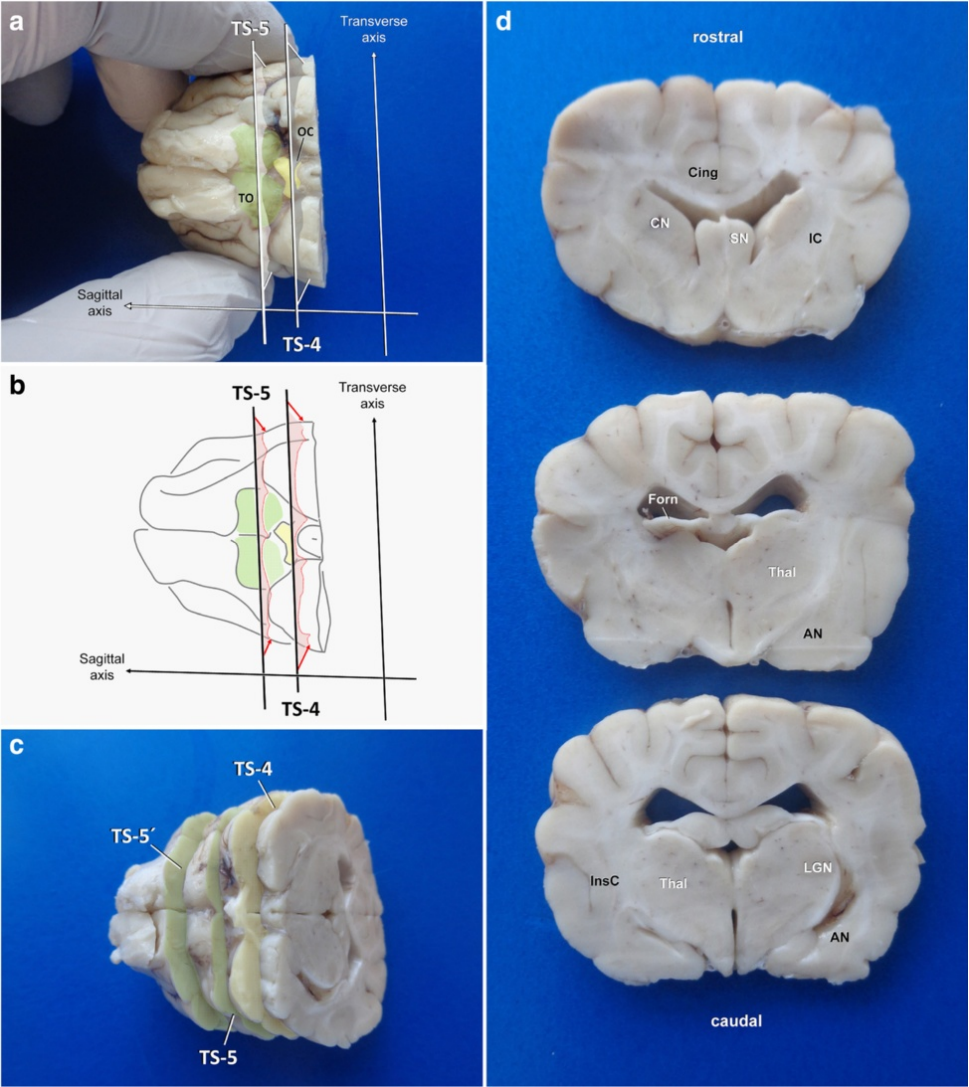
Planning (**a**, **b**) and performance (**c**, **d**) of dissection of the caudal and middle capsular region. AN: amygdaloid nucleus; Cing: cingulate gyrus; CN: caudate nucleus; Forn: fornix; IC: internal capsule; InsC: insular cortex; LGN: lateral geniculate nucleus; OC: optic chiasm; SN: septal nuclei; Thal: thalamus; TO: tuberculum olfactorium

Essential parts of parietal cortex will have already been collected at the thalamic level. For gross inspection, further transverse sections should be performed from ventral at or just proximal of the optic chiasm (**TS-4**; Figs. 3 and 12) to investigate septal nuclei, fornical body, rostral commissure and basal nuclei. Depending on the size of the brain, a parallel transverse section through the middle part of the olfactory tuberculum (**TS-5**) provides a representative view of the frontal lobe, including the caudal parts of frontal cortex, striatum and the capsules (Figs. 3 and 12).

Further trimming of the remaining tissue block B (Table 8) mainly is dedicated to explore motor areas of frontal cortex and the olfactory lobe, which resembles another low threshold area for seizure generation and lesions of which are rarely associated with neurological signs in dogs and cats other than seizures.

**Fig. 13:**
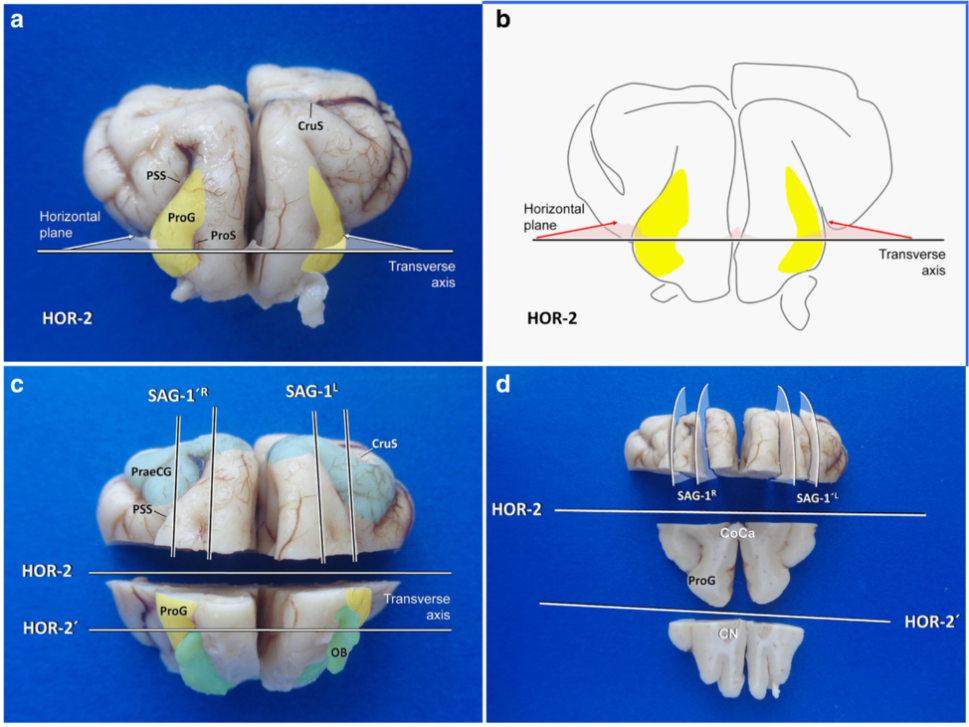
Planning and performance of fronto-olfactory dissection in a dog; rostral view. CN: caudate nucleus; CoCa: corpus callosum. CruS: cruciate sulcus; OB: olfactory bulb; PraeCG: praecruciate gyrus; ProG: proreus gyrus; ProS: prorean sulcus PSS: presylvian sulcus

It proves useful to approach the olfactory bulb and cortex, its connections to the periventricular brain and subventricular zones using horizontal sections. To conduct the first horizontal section (**HOR-2**), the blade is inserted in laterolateral axis at the proreus gyrus and the tissue is cut in rostrocaudal direction (Fig. 13). With the previous transverse cut, set caudal to the genu of the corpus callosum, both hemispheric parts stay connected, which facilitates cutting and processing. Depending on the brain size, one or two further horizontal sections (**HOR-2′, −2″**) are performed at 3–4 mm interslice distances ventral to **HOR-2** (Fig. 13).

Having achieved this, two sagittal sections through the lateral third of the proreus gyrus (**SAG-1Left/Right**) and again about 3 mm lateral to these (**SAG-1′L/R**) allow for inspection of and sampling of motor cortex, flanking the cruciate sulcus rostrally (pre-cruciate) and caudally (post-cruciate) (Fig. 13). Further sagittal sections in vertical plane (**SAG-1″ L/R**) may be taken if for diagnostic purposes.

**Fig. 14:**
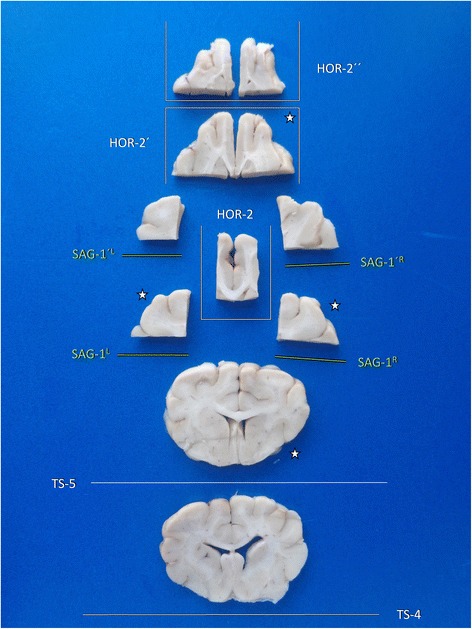
Overview of main brain slabs of Block B in correct angle of section. A selection of these may be further processed for histology. Asterisks mark our recommendation for systematic epilepsy pathology studies

An example of the tissue slabs achieved by trimming of tissue block B is provided in Fig. 14.

### Trimming of the hindbrain (tissue block C)

Brainstem and cerebellar seizures have not been reported in domestic animals yet but there is some histological evidence that epilepsy in dogs maybe associated with cerebellocortical abnormalities [8]. Likewise, cerebellar atrophy is observed in about 25 % of human epileptics presented at autopsy [41] with some variabilities between anterior versus posterior lobe involvement [42]. Cerebellar changes either are related to the seizure-syndrome [8], to antiepileptic drug toxicity [42] or to specific epileptogenic aetiologies, such as hypoxia, ischaemia, intoxication or mitochondrial disease [42, 43]. In contrast, there is no systematic interdependence between epilepsy and brainstem lesions.

Sampling of these areas pretty much underlies laboratory specific protocols with the basic requirement to obtain sections from the cerebellum in two planes and to investigate vital brainstem centres (Table 9).

In the following, one possible approach is illustrated which, based upon the experience gained at our own laboratories (LMU Munich, UAB Barcelona), has proven easy to perform and to standardise and is effective in picking-up lesions blindly.

#### Procurement of mesencephalon

After **TS-2**, a transversely oriented tissue section is taken from the caudal mesencephalic stump, either at the intercollicular level or the level of the rostral colliculi (**TS-2′**). The caudal colliculi are sampled later on via paramedian sagittal sections in vertical plane (see below).

#### Procurement of cerebellum and medulla oblongata at mid-cerebellar level

**Fig. 15:**
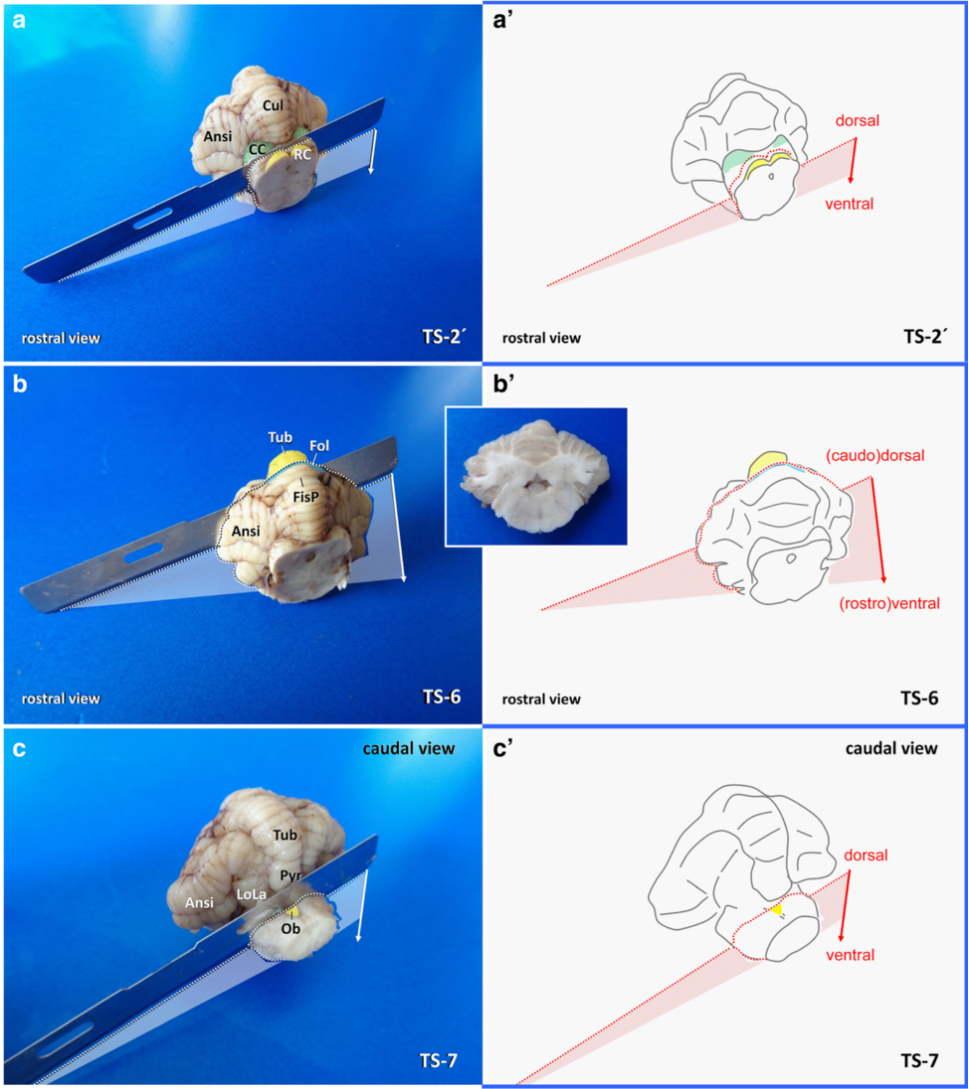
Dissection of central midbrain (**a**, **a´**), central metencephalon (**b**, **b´**) and obex area (**c**, **c´**) in three steps. Ansi: ansiforme lobule; CC: caudal colliculus; Cul: culmen; FisP: fissura prima; Fol; folium; LoLa: lateral lobules; Ob: obex; Pyr: pyramis; RC: rostral colliculus.; Tub: tuber. Canine brain

In order to obtain a representative transverse section, the cerebellum is approached from dorsal. After mesencephalic sampling, sectioning (**TS-6**; Fig. 15) is carried out in a dorsoventral direction along the dorsoventral axis of the cerebellar hemispheres, with the long blade being inserted 2–3 mm caudal to the primary fissure. The parallel section (**TS-6′**), necessary to obtain a tissue slice is then performed either on the rostral or caudal stump, depending on the placement of the cerebellar roof nuclei (Additional file 1).

This section provides a detailed view on the flocculonodular lobe, paraflocculus, paravermis and dorsal vermis, the cerebellar roof, including the associated nuclei, the caudal peduncles or lateral foramina, and the medulla at its largest laterolateral diameter that contains in particular the dorsolateral sensory nuclei and motor nuclei of CN-VI and CN-VII (Fig. 15).

#### Procurement of the caudal vermis and the autonomic centres of the caudal brainstem

Even though the last section being broadly considered representative of the cerebellum, it does not contain the essential spinocerebellar parts of the vermis, since the nodulus belongs to the vestibulocerebellum and the dorsal aspects of the vermis receive cortico-ponto-cerebellar inputs. Furthermore, the medulla being cut at mid rostocaudal level does not contain the respiratory control centre. In particular in combined (medullocerebellar) midline pathologies, such as in transforaminal cerebellar herniation [29], it is essential to study the micromorphology of these areas in detail.

Most of the vagal nerve nuclei and related parasympathetic nuclei are preserved by gathering a transversely oriented slab of brainstem from the obex area (**TS-7**; Fig. 15).

**Fig. 16:**
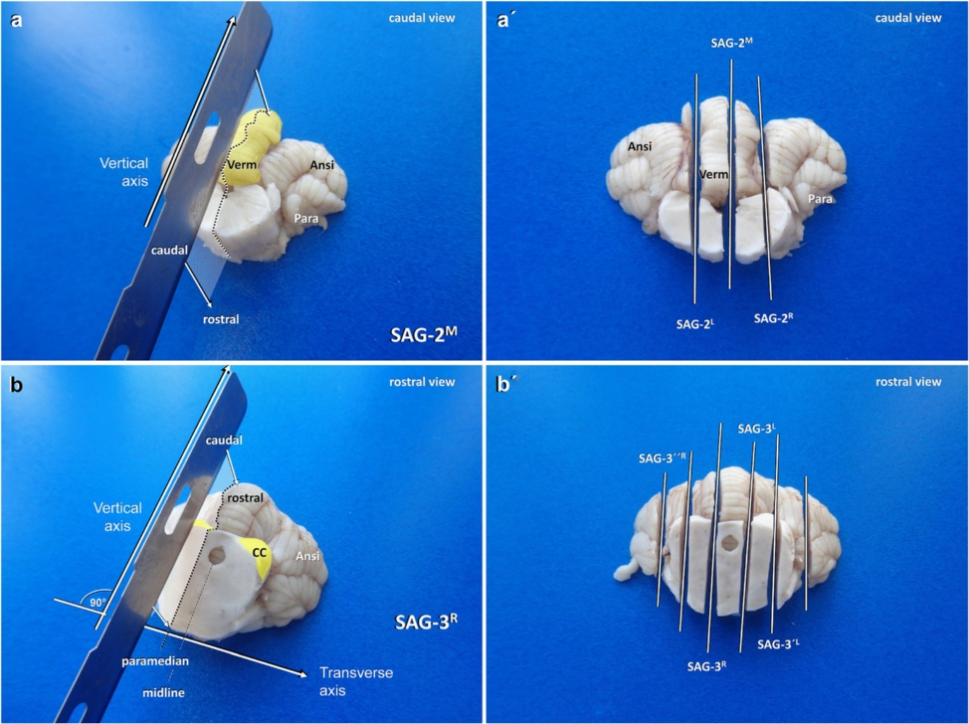
Sagittal dissection of the caudal (**a**, **a´**) and rostral (**b**, **b´**) cerebellar lobes and the associated brain stem in a dog. Ansi: ansiform lobule; CC: caudal colliculus; Para: paraflocculus; Verm: vermis

After that, the caudal part of the cerebellum and brain stem can be sectioned sagittally through the midline (**SAG-2 M**) and in sequential paramedian slides (**SAG-2′L/R**; Fig. 16).

Histological slides from these brain slices allow for inspection of the comb-like two dimensional organisation of the Purkinje cell dendrites, which is not possible on transverse sections. It further elucidates histopathological sequelae of transtentorial herniation, which may be subtle and restricted to the lingula or pyramis.

#### Obtainment of rostral cerebellar lobe and caudal mesencephalon

Concerning, the transtentorial border zone, the implied brain shifting and associated problems, the cerebellum may have suffered from descending occipital lobes. In contrast to transforaminal herniation, caudal transtentorial protrusion of the occipital lobes results in a lesion of the paravermal areas of the rostral cerebellum [29]. Midline sections, hence, do not necessarily reflect the effects of impingement. Evaluation of the rostral lobe further may pick-up the anterior type of epilepsy-related cerebellar atrophy [42].

Investigation of the brainstem underlying the rostral cerebellar lobe, on the other hand, could help to detect systemic ictogenic conditions such as global ischaemia [44]

There are two different assessment modes that may be applied, depending on the individual case scenario. The easier procedure (Table 9, Fig. 16) employs two parallel sagittal or slightly inwardly rotated paramedial sections in the rostrocaudal direction through caudal colliculi and/or rostral peduncles (CC/RP) and the caudally adjacent paravermis (**SAG-3 L/R**) as well as parallel sections (**SAG-3′L/R**) conducted 3 mm farther lateral (Fig. 16).

**Fig. 17:**
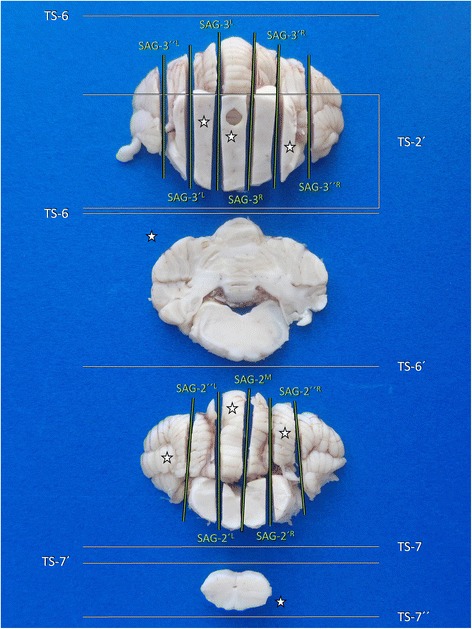
Overview of main brain slabs of Block C in correct angle of section. A selection of these may be further processed for histology. Asterisks mark our recommendation for systematic epilepsy pathology studies

Figure 17 provides a summary of the possible tissue slabs generated through the described protocol for tissue block C trimming (Table 9).

An alternative option, used in distinct rostral compression of the cerebellum would be a horizontal section of the cerebellum just dorsal to the colliculi with subsequent bilateral sagittal sections through the “decapitated” CC/RP.

## Post-Trimming procedures and histological staining

Independent of the sections necessary for the requested diagnosis, processing of the brain sections to paraffin blocks is advisable to prevent the brain tissue from formalin-induced, excessive aldehyde bridging and DNA fragmentation. Processing cycles vary slightly in between different labs and run on standard or, even better, dedicated CNS programs with or without dimethylsulfoxide permeabilisation [45]. Table 10 provides an example of a CNS adapted paraffin embedding cycle. It has to be made clear that any attempt to accelerate histoprocessing will impact negatively on the tissue quality and thereby compromise detection of degenerative cytopathological features. Identification of infiltrative changes will be less severe.

**Table 10:** Example of a CNS specific processing/embedding cycle [45]

Incubation time	Chemical	Temperature
6 h	70 % ethanol	40 °C
4 h	80 % ethanol	40 °C
4 h	90 % ethanol	40 °C
4 h	100 % ethanol	40 °C
4 h	100 % ethanol	40 °C
4 h	100 % ethanol	40 °C
2 h	xylene	40 °C
2 h	xylene	40 °C
2 h	xylene	40 °C
1 h	paraffin^a^	60 °C
1 h	paraffin^a^	60 °C
1 h	paraffin^a^	60 °C
3 h	paraffin^a^	60 °C

Cycle times differ if fixation is performed with higher concentrations of formalin as occasionally recommended for large specimens

^a^We recommend use of paraffin with 4 % DMSO (Paraplast®, Leica Biosystems, Nussloch)

Staining protocols, in addition to haematoxylin-eosin (H.E.), are to be chosen in accordance to (1) the requirements of the individual case, (2) the investigational purpose and (3) financial constraints. Overviews on neuropathological standard stains are provided elsewhere [46]

For elucidation of epilepsy-related changes it proved beneficial to highlight the regional drop-out of nerve cells by cresyl violet-based stains such as Nissl stain (without myelin staining) or Kluver Barrera stain (with myelin staining). In very fresh samples taken via brain surgery or early post-mortem, NeuN immunohistochemistry may be superior for highlighting neurons [47] but this procedure also is far more expensive and immunoreactivity rapidly decreases post-mortem and with prolonged fixation periods.

Apart from providing an insight into nerve cell density neuronal stainings also facilitate the detection of histoarchitectural grey matter changes, such as dyslamination, and heterotopia [47]. Dysmorphic neurons, on the other hand, become most obvious on staining for microtubule associated protein 2 (MAP-2) and neurofilament staining. Just the interpretation requires some experience in neuronal cytoarchitecture [47].

In post-mortem samples, differentiation of post- and intra-ictal neuronal necrosis from terminal ischaemic changes can be problematic, in particular if prefinal seizure episodes might have gone unseen. In such cases, clarification of the fate of eosinophilic neurons can be achieved using FluoroJade-B® or -C® [48, 49]. Other, more specific markers of degeneration, necrosis and apoptosis may be used based on the aim of investigation and the experiences of the investigator.

Experience also comes into effect with evaluation of glial response. Reactive astroglial changes occur with or without preceding neuronal degeneration. Protoplasmatic astrogliosis may be missed if the examiner is not familiar with astroglial cytomorphological details. It becomes even more sophisticated to identify fibrillary astrogliosis and isomorphic astrocytosis, without cytoplasmic accumulation. Intraobserver’s sensitivity can be increased for both fibrillary and protoplasmic astrogliosis by staining for the filament glial fibrillary acidic protein (GFAP) and by use of the overall available marker vimentin [17].

Most recently, the role of autoimmune mechanisms [14] and neuroinflammation have gained new attention in veterinary epileptology and led to introduction of immunosuppressive and anti-inflammatory treatment concepts [50]. With regards to autoimmune encephalitis, conventional markers for lymphocyte subsets, antibodies and complement factors may shed light on their specific involvement [14], while cellular infiltrates are seen on standard stains (e.g. H.E.).

With ionized calcium-binding molecule (Iba1), even subtle changes to the microglial activity can be nicely visualised in paraffin embedded tissues of different animal species [51] including the hippocampi of dogs [52]. In combination with CD-163, It has also proved to be a reliable marker for distinction of local microglial response and invasive macrophages in canine encephalitis [53].

Breakdown of the blood brain barrier due to seizures or their primary pathologies will lead to pervasive effects due to extravasation of fluid and possibly epilepsy promoting molecules [54]. Postictal brain oedema usually is quite prominent and its extension into the white matter remains visible for a prolonged period with proper brain processing (see above). In grey matter, however, reabsorption is quick and an oedema diagnosis may require staining for the water channel molecule aquaporin 4 [55]. As surrogate for the possible influx of neuroactive agents immunohistochemical staining for albumin may be performed [54]

The list of histological tools could be further extended. The major diagnostic purpose, though, is to identify epileptogenic and postictal changes and to shed light on possibly epileptogenic pathologies. It rarely is the staining panel that limits the success of brain histology in clinical patients. Instead the relevant area may be easily missed. For most investigations, H.E. staining combined with Nissl’s stain and GFAP will provide sufficient data for the clinician.

## What the pathologist should know about the case?

Pathological studies on epilepsy brains in animals mainly aim to identify undiagnosed seizure aetiologies, comorbidities and the substrate of drug-resistance as well as to relate clinical findings, including the focality of seizures, to morphological changes.

For a meaningful investigation, a certain data set has to be obtained from the veterinarian and/or the owner (Table 11) that clarifies predisposing factors and pedigree data, the possibility of preceding or precipitating events, possible exposure to toxins, neurological signs, phenomenology and time course of the paroxysmal disorder, MRI and EEG data, concurrent medical problems and therapy response.

**Table 11:** Essential data (Level I) that are required to be collected for a meaningful post-mortem examination

I. Data on the animal and pedigree	# breed, age, gender
	# evidence of seizures or other paroxysmal and neurological diseases in the pedigree
	# usual food and treats, dietary changes
	# exposure to toxins/medications
II. Data on the events & clinical presentation	# possible triggers
	# seizure onset semiology and characteristics
	# evidence of automatisms
	# interictal neurological signs/neurolocalisation
	# seizure frequency and duration
	# abnormal MRI and EEG findings
	# abnormalities on blood work, CSF and urine analysis
III. Epicrisis	# treatment scheme and response
	# changes to semiology
	# recently acquired medical problems
	# time span from last seizure to death
	# natural death or euthanasia
	# death in status epilepticus

Clinical data can be stratified, as Level 1 data (basic) that are mandatory and Level 2 data (detailed) that are optional. The questionnaires very much benefit from requesting as much objective and binary parameters as possible.

If not even Level 1 data can be obtained, efforts should not be wasted, since pathological findings are not able to produce and replace clinical observations. Those patients must not be included in scientific studies as neither impact nor relevance of tissue findings can be reproduced. The same holds true for acquisition of control animals. Seizure freedom has to be sought with the same stringency as seizure histories in epilepsy patients.

## Conclusions and outlook

Epilepsy is a highly prevalent disease in veterinary practice that demands to be investigated using a multi- and transdisciplinary approach. Unfortunately, brain pathology has been broadly perceived as a confirmative rather than investigative tool in the retrospective work-up of epileptic companion pets. This lack of enthusiasm may be due to the paucity of tissue changes even in severe clinical presentations [56], the sometimes overwhelming severity of non-specific ictal and postictal changes, and the elusive ambition to localise an epileptic focus in the huge brain without EEG and functional imaging data or a thorough sampling scheme.

Even though the advances in human epileptology are dominated by the activities on focal epilepsy, we may profit from the experiences in those cases and from paradigms that were brought to light by studies in rodents. In fact, natural epilepsy in dogs and cats resembles an ideal playground to test hypotheses originating from “mice and men”. Comparative neuropathological concepts, indeed, have unravelled important pathobiological data that may impact on the clinical management and prognostic considerations of epileptic animals [13, 14].

It remains to be seen that in animals advances in EEG, functional imaging and brain surgery will translate into surgical removal of epileptogenic brain tissue, other than lesionectomy [1]. Until then we should benefit from the availability of post-mortem brains the offer a precious opportunity to study anatomical, neurochemical and molecular determinants for seizure progression and drug resistance, if the tissue has been stored and processed accurately and changes, on high resolution, can be attributed to specific functional brain regions. By application of the procedures illustrated herein the caseload of epilepsies of unknown cause may be further narrowed [57, 58].

Most hitherto published tissue studies in dogs and cats, however, underscore even baseline neuroanatomical accuracy and lack reproducible sampling schemes. That way, the relevance of published findings for a larger population of epileptic animals remains obscure, at best.

Even if the investigations may be high pitched and restricted to specialised laboratories, accurate sampling of epileptic brains can be performed at virtually any place with minimal training requirements. The true impact of the studies, on the other hand, very much depends on these, less appreciated early investigational steps.

Since the mission of this group is to foster diagnosis, research and clinical care of epilepsy in companion animals, this paper aims to ensure efficient brain sampling by pathologists and neurologists. The above described guideline rather has been tested in untrained staff and rapidly can be implemented into every pathology laboratory that wishes to contribute to the alliance against epilepsy.

## Additional file

Additional file 1:
**Video featuring a real time demonstration of systematic brain trimming.** Please note, that other approaches may be necessary for distinct lesions and neurological signs.

## Abbreviations

AN: Amygdaloid nucleus
Ansi: Ansiform lobule
Aqu: Mesencephalic aqueduct
ARAS: Ascending reticular activating system
BA: Basilar artery
CA: Cornu ammonis
CC: Caudal colliculus
CCG: Caudal composite gyrus
Cing: Cingulate gyrus
Cla: Claustrum
CN: Caudate nucleus
CN-III/-VI/-VII: Cranial nerves III/VI/VII
CNS: Central nervous system
CoCa: Corpus callosum
Cru: Crura cerebri
CruS: Cruciate sulcus
Cul: Culmen
CV: Caudal vertex
DM: Dura mater
DNA: Deoxyribonucleic acid
EEG: Electroencephalography
ERC: Entorhinal cortex
FFPE: Formalin-fixed paraffin embedded
FISS: Fissures, interfoliar spaces, sulci
FisP: Primary fissure
Fol: Folium
Forn: Fornix
GFAP: Glial fibrillary acidic protein
HC: Hippocampal commissure
HE: Haematoxylin eosin
HOR: Horizontal section
HS: Hippocampal sclerosis
IC: Internal capsule
IF: Intercrural fossa
ILAE: International League Against Epilepsy
InsC: Insular cortex
IVETF: International Veterinary Epilepsy Task Force
LGN: Lateral geniculate nucleus
LoLa: Lateral lobule
LRS: Lateral rhinal sulcus
Mam: Mammillary bodies
MAP: Microtubule-associated protein
MCA: Middle cerebral artery
MRI: Magnetic resonance imaging
OB: Olfactory bulb
Ob: Obex
OC: Optic chiasm
PAG: Periaqueductal gray matter
Para: Paraflocculus
ParaH: Parahippocampal gyrus
PeRC: Perirhinal cortex
OV: Occipital vertex
PiLo: Piriform lobe
Pit: Pituitary stalk
PL: Pathological lesion
PoRC: Postrhinal cortex
PPC: Prepiriform cortex
PraeCG: Praecruciate gyrus
ProG: Prorean gyrus
ProS: Prorean sulcus
PSS: Presylvian sulcus
Pyr: Pyramis
RC: Rostral colliculus
RP: Rostral peduncle
SAG: Sagittal section
SN: Spetal nuclei
SO: Stria olfactoria
SOB: Supraoccipital bone
SplG: Splenial gyrus
SUDEP: Sudden Unexpected Death in Epilepsy
TB: Trimmed brain
TFOP: Transverse fibres of pons
Thal: Thalamus
TILT: Tilted section
TO: Tuberculum olfactorium
Tra: Trapezoid body
TS: Transverse section
Tub: Tuber
TVB: Temporoventral body
UB: Unfixed brain
Uv: Uvula
Verm: Vermis
VC: Visual cortex
